# Clinical outcomes of hospitalized COVID-19 patients treated with remdesivir: a retrospective analysis of a large tertiary care center in Germany

**DOI:** 10.1007/s15010-022-01841-8

**Published:** 2022-05-12

**Authors:** Kathrin Marx, Ksenija Gončarova, Dieter Fedders, Sven Kalbitz, Nils Kellner, Maike Fedders, Christoph Lübbert

**Affiliations:** 1 Hospital Pharmacy, Hospital St. Georg, Delitzscher Str. 141, 04129 Leipzig, Germany; 2 Department of Radiology, University Hospital Carl Gustav Carus, Fetscherstr. 74, 01307 Dresden, Germany; 3 Department of Infectious Diseases/Tropical Medicine, Nephrology and Rheumatology, Hospital St. Georg, Delitzscher Str. 141, 04129 Leipzig, Germany; 4 Division of Infectious Diseases and Tropical Medicine, Department of Medicine II, Leipzig University Hospital, Liebigstr. 20, 04103 Leipzig, Germany; 5 Interdisciplinary Center for Infectious Diseases, Leipzig University Hospital, Liebigstr. 20, 04103 Leipzig, Germany

**Keywords:** SARS-CoV-2, COVID-19, Treatment, Remdesivir, Corticosteroids, Outcome, Mortality

## Abstract

**Purpose:**

The benefits of antiviral treatment with remdesivir in hospitalized patients with COVID-19 remain controversial. Clinical analyses are needed to demonstrate which patient populations are most likely to benefit.

**Methods:**

In a retrospective monocentric analysis, patients with COVID-19 treated between July 1, 2020 and June 30, 2021 at Hospital St. Georg, Leipzig, Germany were evaluated. The primary endpoint was time to clinical improvement, and the secondary endpoint was 28-day mortality. Propensity score matching was used for the endpoint analysis.

**Results:**

A total of 839 patients were fully evaluated, 68% of whom received specific COVID-19 drug therapy. Remdesivir was used in 31.3% of the patients, corticosteroids in 61.7%, and monoclonal antibodies in 2.3%. While dexamethasone administration was the most common therapeutic approach during the second pandemic wave, combination therapy with remdesivir and corticosteroids predominated during the third wave. Cox regression analysis revealed that combination therapy was not associated with faster clinical improvement (median: 13 days in both matched groups, HR 0.97 [95% CI 0.77–1.21], *P* = 0.762). By contrast, 28-day mortality was significantly lower in the corticosteroid-remdesivir group (14.8% versus 22.2% in the corticosteroid group, HR 0.60 [95% CI 0.39–0.95], *P* = 0.03) in the low-care setting. This effect was also demonstrated in a subgroup analysis of patients with remdesivir monotherapy (*n* = 44) versus standard of care (SOC).

**Conclusion:**

In COVID-19 patients with only mild disease (low-flow oxygen therapy and treatment in a normal ward) who received corticosteroids and/or remdesivir in addition to SOC, early administration of remdesivir was associated with a measurable survival benefit.

**Supplementary Information:**

The online version contains supplementary material available at 10.1007/s15010-022-01841-8.

## Introduction

Severe acute respiratory syndrome coronavirus type 2 (SARS-CoV-2) was first identified as the cause of a respiratory illness outbreak in Wuhan, Hubei Province, China, in December 2019. The associated disease was named *coronavirus disease 2019* (COVID-19). Starting from a local outbreak, SARS-CoV-2 spread rapidly worldwide, leading the World Health Organization (WHO) to declare COVID-19 a pandemic on March 11, 2020 [1]. As of February 20, 2022, more than 418 million COVID-19 cases and 5.8 million deaths have been reported to WHO worldwide [2]. In Germany, more than 13.2 million people have contracted COVID-19, and more than 120,000 people have died as of February 20, 2022 [3]. Therefore, the optimal implementation of therapeutics to reduce COVID-19 morbidity and mortality has become a global priority.

In this context, the new broad-spectrum antiviral remdesivir, a ribonucleotide analogue that inhibits viral RNA polymerase, has received considerable attention and has been the subject of controversial recommendations: Based on the results of the NIAID-ACTT-1 trial [4], the European Medicines Agency (EMA) concluded that the benefits of remdesivir in COVID-19 patients with pneumonia who require supplemental oxygen (low- or high-flow oxygen therapy or other noninvasive ventilation) outweigh the risks, and that the drug could be approved in the European Union under *special conditions*; by contrast, the WHO, in its guideline [5] based on the SOLIDARITY trial [6], advises against the use of remdesivir in hospitalized patients with COVID-19. The European Respiratory Society (ERS) and the Association of the Scientific Medical Societies in Germany (AWMF) did not advocate for or against remdesivir therapy in their treatment guidelines because of continuing uncertainties about its potential benefits [7–9]. The results of the RECOVERY trial [10] led WHO and EMA to recommend low-dose dexamethasone in COVID-19 patients [5, 11]. A post-hoc analysis of the final ACTT-1 publication showed the treatment benefits of remdesivir and glucocorticoids in a subgroup, leading the authors to suggest a possible additive effect [4]. Based on the theoretical combined benefits of antiviral and anti-inflammatory therapies, the National Institutes of Health (NIH) recommended the combination of dexamethasone plus remdesivir as a treatment option for patients requiring supplemental oxygen (low- or high-flow oxygen therapy or other non-invasive ventilation) [12]. In its recommendation on pharmacotherapy for COVID-19, the German Society for Infectious Diseases (DGI) refers to the combination of remdesivir plus dexamethasone in severe and critical illnesses with supplemental oxygen support, citing the lack of clinical data on combination therapy [13]. Finally, the ongoing NIAID-ACTT-4 trial [14] is evaluating the combination of baricitinib, a selective and reversible janus kinase inhibitor, and remdesivir compared with dexamethasone-remdesivir combination therapy; however, results are not yet available.

This retrospective study of treatment options for hospitalized adults with COVID-19, which focused on the therapeutic effects of remdesivir, was designed to provide a center-based evaluation of established therapies for COVID-19. Specifically, the outcomes of patients receiving corticosteroid-remdesivir combination therapy were compared with those of patients receiving corticosteroids alone, and the outcomes of patients receiving remdesivir monotherapy were compared with those of patients receiving standard supportive care (SOC) alone. A clinical and demographic analysis of COVID-19 patients was also performed, distinguishing between the second (October 2020 to February 2021) and third (March to May 2021) COVID-19 waves in Germany.

## Patients and methods

### Setting

The Hospital St. Georg in Leipzig, Saxony, Germany is a large tertiary-care hospital with 1,066 beds and 25 different specialist areas and clinics embedded in the structure of a modern academic teaching hospital. Patients with COVID-19 are primarily cared for in two infectious disease units, with a total of 44 beds.

### Study design and participants

In this retrospective analysis, all adult patients (≥ 18 years) with polymerase chain reaction-confirmed SARS-CoV-2 infection who were hospitalized between July 1, 2020, and June 30, 2021 were included. The following patient data were recorded: age, sex, body mass index (BMI), heart rate, blood pressure, respiratory rate, date of admission and discharge, admitting and discharging ward, principal and secondary diagnoses, case-mix index, type of infection (nosocomial or community acquired), specific COVID-19 therapy, and laboratory parameters on admission (leukocyte count, platelet count, creatinine, C-reactive protein, alanine aminotransferase, aspartate aminotransferase, bilirubin, D-dimer, interleukin-6, lactate dehydrogenase, procalcitonin).

The disease severity of patients with COVID-19 was staged according to the WHO Clinical Progression Scale with eight categories [15]: (1) ambulatory without limitation of activity, (2) ambulatory with limited activity, (3) hospitalized without oxygen therapy, (4) hospitalized on oxygen therapy by mask or nasal prongs, (5) hospitalized receiving non-invasive ventilation, (6) hospitalized with intubation and mechanical ventilation, (7) hospitalized with mechanical ventilation and additional organ support, such as vasopressors, renal replacement therapy, or extracorporeal membrane oxygenation, and (8) death.

### Ethics approval

The study was conducted in accordance with the ethical guidelines of the 1964 Declaration of Helsinki and its later amendments and was approved by the local ethics committee (Saxonian Board of Physicians, Dresden, Germany, vote EK-BR-65/21–1).

### Clinical endpoints

The primary endpoint was time to clinical improvement (discharge from hospital) within 28 days. The absence of clinical improvement was censored after 28 days. The secondary end point was time to death within 28 days.

### Timeline of the COVID-19 pandemic in Germany

According to the course of infection events in Germany, the COVID-19 pandemic was divided into individual phases that are analogous to the phase classification of the Robert Koch Institute (RKI) [16]: Summer plateau 2020 (calendar weeks 21–39, 2020), second COVID-19 wave (calendar week 40, 2020 to calendar week 8, 2021), third COVID-19 wave (calendar weeks 9–23, 2021), and summer plateau 2021 (calendar weeks 24–37, 2021).

### Statistical analysis

Propensity score (PS) matching was used to account for the collected characteristics of treated and untreated patients, similar to a randomized controlled trial (RCT) [17, 18]. The PS was estimated from the available data using a logistic regression model in which therapy was the dependent variable, and patient characteristics existing at the start of therapy served as the independent variables (age, sex, BMI, laboratory parameters on the day of admission, and diagnoses). In PS matching, each treated patient was assigned an untreated patient with the same PS (1:1 matching) or a minimally different PS. The standardized mean difference was used to show the differences between the different groups. For the descriptive comparison of the survival times of PS-matched cases, the Kaplan–Meier procedure was used. The log-rank test served as a significance test for comparison between the two studied groups, and the hazard ratio (HR) served as a descriptive measure of the difference in survival times. Data analysis of patient characteristics was performed using the chi-square test for categorical variables and t tests for continuous variables. Statistical analyses were performed using R statistical software (version 4.1.2) with implementation of the PS package (MatchIt) [19].

## Results

### Patients

**Fig. 1:**
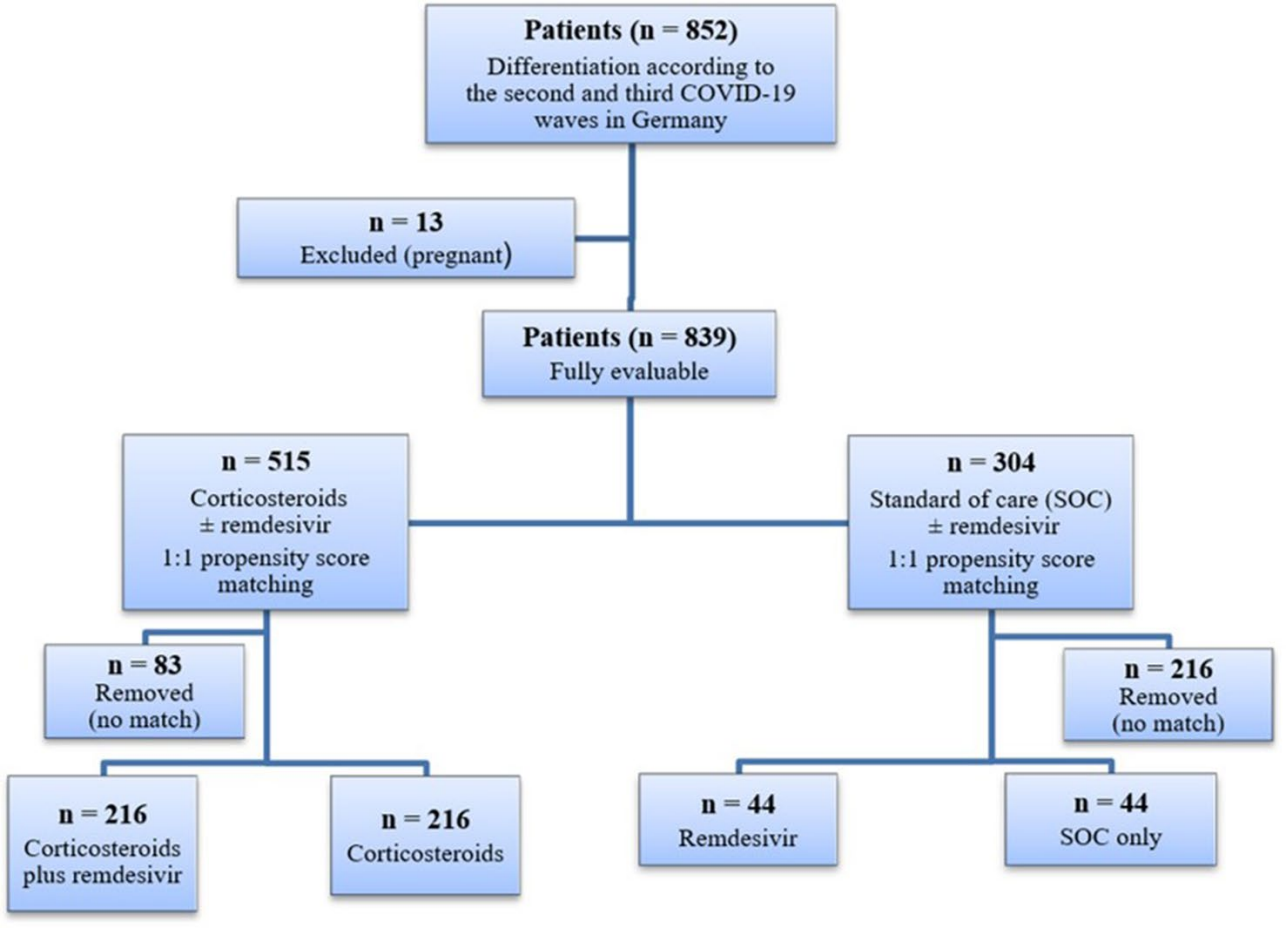
Flowchart of the study population

Within the observation period, 852 cases were retrospectively reviewed; 13 pregnant women were excluded from the outcome analysis. Of the remaining 839 patients, 515 received corticosteroids (primarily dexamethasone) either as monotherapy or in combination with remdesivir, 260 patients received no additional COVID-19 drug therapy (apart from SOC), and 44 patients received remdesivir as monotherapy (Fig. 1).

The majority of COVID-19 patients were admitted between calendar week 40, 2020 and calendar week 23, 2021. As only a few cases were hospitalized in the respective summer plateaus, the second and third COVID-19 waves of the pandemic are the focus of our evaluation. Table 1 shows the baseline characteristics of the 852 cases and their distribution during the second and third COVID-19 waves.

**Table 1:** Baseline characteristics of in-patients with COVID-19 treated at Hospital St. Georg in the period from July 1, 2020 to June 30, 2021

Characteristics	Second COVID-19 wave	Third COVID-19 wave	*P* value	SMD	All patients
Number of patients (*n*)	526	291			852
Age (mean, SD)	72.13 (15.26)	66.14 (17.88)	< 0.001	0.361	69.4 (16.8)
*Age group* (%)					
18–34 years	17 (3.2)	22 (7.6)	< 0.001	0.393	45 (5.3)
35–59 years	76 (14.4)	70 (24.1)			161 (18.9)
60–79 years	222 (42.2)	125 (43.0)			355 (41.7)
≥ 80 years	211 (40.1)	74 (25.4)			291 (34.2)
*Sex* (%)					
Male	295 (56.1)	172 (59.1)	0.446	0.061	488 (57.3)
Female	231 (43.9)	119 (40.9)			364 (42.7)
BMI (mean, SD)	28.51 (5.80)	29.32 (6.82)	0.147	0.128	28.83 (6.20)
CMI (mean, SD)	1.65 (2.86)	1.42 (1.84)	0.218	0.095	1.58 (2.52)
Length of stay (mean, SD)	14.74 (13.94)	12.46 (9.68)	0.014	0.190	13.80 (12.48)
*Disease severity* (%)					
Mild disease (no oxygen therapy), WHO score 3	118 (22.4)	52 (17.9)	0.006	0.303	185 (21.7)
Mild disease (oxygen by mask or nasal prongs), WHO score 4	224 (42.6)	140 (48.1)			370 (43.4)
Severe disease (non-invasive ventilation or high-flow oxygen), WHO score 5	60 (11.4)	50 (17.2)			123 (14.4)
Severe disease (intubation and mechanical ventilation), WHO score 6	8 (1.5)	8 (2.7)			16 (1.9)
Severe disease (ventilation and additional organ support, vasopressors, RRT, ECMO), WHO score 7	3 (0.6)	0 (0.0)			3 (0.4)
Death (%)	113 (21.5)	41 (14.1)			155 (18.2)
*Ward type* (%)					
Intensive care unit (ICU)	118 (22.4)	51 (17.5)	0.117	0.123	171 (20.1)
Normal ward	408 (77.6)	240 (82.5)			681 (79.9)
*Type of infection* (%)					
Community-acquired	468 (89.0)	269 (92.4)	0.141	0.120	772 (90.6)
Nosocomial	58 (11.0)	22 (7.6)			80 (9.4)
Respiratory rate/min (SD)	21.31 (6.03)	20.95 (5.54)	0.487	0.063	21.02 (5.86)
*Laboratory results (mean, SD)*					
Estimated glomerular filtration rate (GFR, mL/min/1.73 m^2^)	65.95 (33.70)	77.74 (33.61)	< 0.001	0.350	70.81 (34.47)
C-reactive protein (CRP, mg/L)	80.13 (103.55)	74.01 (70.02)	0.382	0.069	76.65 (92.07)
Lactate dehydrogenase (LDH, mmol/L*s)	5.77 (3.10)	5.85 (2.43)	0.768	0.028	5.76 (2.84)
Leukocyte count (Gpt/L)	8.28 (9.32)	6.55 (3.18)	0.002	0.249	7.63 (7.66)
Platelet count (Gpt/L)	216.39 (106.43)	197.60 (90.64)	0.012	0.190	209.57 (100.85)
ALAT (µmol/L*s)	0.66 (0.77)	0.56 (0.36)	0.085	0.171	0.62 (0.65)
ASAT (µmol/L*s)	1.01 (1.25)	0.84 (0.55)	0.070	0.174	0.93 (1.04)
D-dimer (µg/L)	2756.57 (4272.95)	2114.04 (3880.60)	0.063	0.157	2468.45 (4087.43)
*Diagnoses* (%)					
Hypertension	377 (71.7)	180 (61.9)	0.005	0.210	573 (67.3)
Cardiovascular disease	421 (80.0)	193 (66.3)	< 0.001	0.313	631 (74.1)
Diabetes	182 (34.6)	72 (24.7)	0.005	0.217	260 (30.5)
Obesity	75 (14.3)	52 (17.9)	0.207	0.098	131 (15.4)
Chronic kidney disease	188 (35.7)	66 (22.7)	< 0.001	0.290	263 (30.9)
Liver disease	12 (2.3)	7 (2.4)	1000	0.008	19 (2.2)
Cancer	53 (10.1)	26 (8.9)	0.685	0.039	81 (9.5)
Asthma	18 (3.4)	19 (6.5)	0.062	0.143	39 (4.6)
COPD	54 (10.3)	25 (8.6)	0.514	0.057	81 (9.5)
Dementia	83 (15.8)	12 (4.1)	< 0.001	0.397	96 (11.3)
Delirium	32 (6.1)	10 (3.4)	0.140	0.125	43 (5.0)
*Charlson Comorbidity Index* (CCI)					
Score 0 (%)	74 (14.1)	83 (28.5)	< 0.001	0.376	173 (20.3)
Score 1–4 (%)	422 (80.2)	200 (68.7)			641 (75.2)
Score ≥ 5 (%)	30 (5.7)	8 (2.7)			38 (4.5)
*Onset of symptoms*					
Onset of symptoms before hospitalization in days (mean, SD)	4.87 (4.51)	5.63 (4.28)	0.067	0.174	5.16 (4.35)
Onset of symptoms before initiation of remdesivir therapy in days (mean, SD)	5.25 (2.8)	4.96 (2.67)	0.484	0.106	5.12 (2.69)
*COVID-19 drug therapy* (%)					
No specific therapy / SOC only	206 (39.2)	51 (17.5)	< 0.001	0.725	273 (32.0)
Corticosteroids	203 (38.6)	94 (32.3)			299 (35.1)
Corticosteroids, mAbs	0 (0.0)	5 (1.7)			5 (0.6)
Remdesivir	24 (4.6)	13 (4.5)			44 (5.2)
Remdesivir, corticosteroids	91 (17.3)	116 (39.9)			216 (25.4)
Remdesivir, corticosteroids, mAbs	0 (0.0)	5 (1.7)			6 (0.7)
Remdesivir, mAbs	1 (0.2)	0 (0.0)			1 (0.1)
mAbs	1 (0.2)	7 (2.4)			8 (0.9)
Initiation of remdesivir therapy ≤ 7 d after hospitalization	114 (99.1)	134 (100.0)	0.939	0.132	266 (99.6)

*ALAT *alanine aminotransferase, *ASAT *aspartate aminotransferase, *BMI* body mass index, *CMI* case-mix index, *RRT* renal replacement therapy, *ECMO* extracorporeal membrane oxygenation, *D-dimer* dimerized plasmin fragment D, *COPD* chronic obstructive pulmonary disease, *mAbs* monoclonal antibodies, *SD* standard deviation, *SMD* standardized mean difference, *SOC* standard of care

**Fig. 2:**
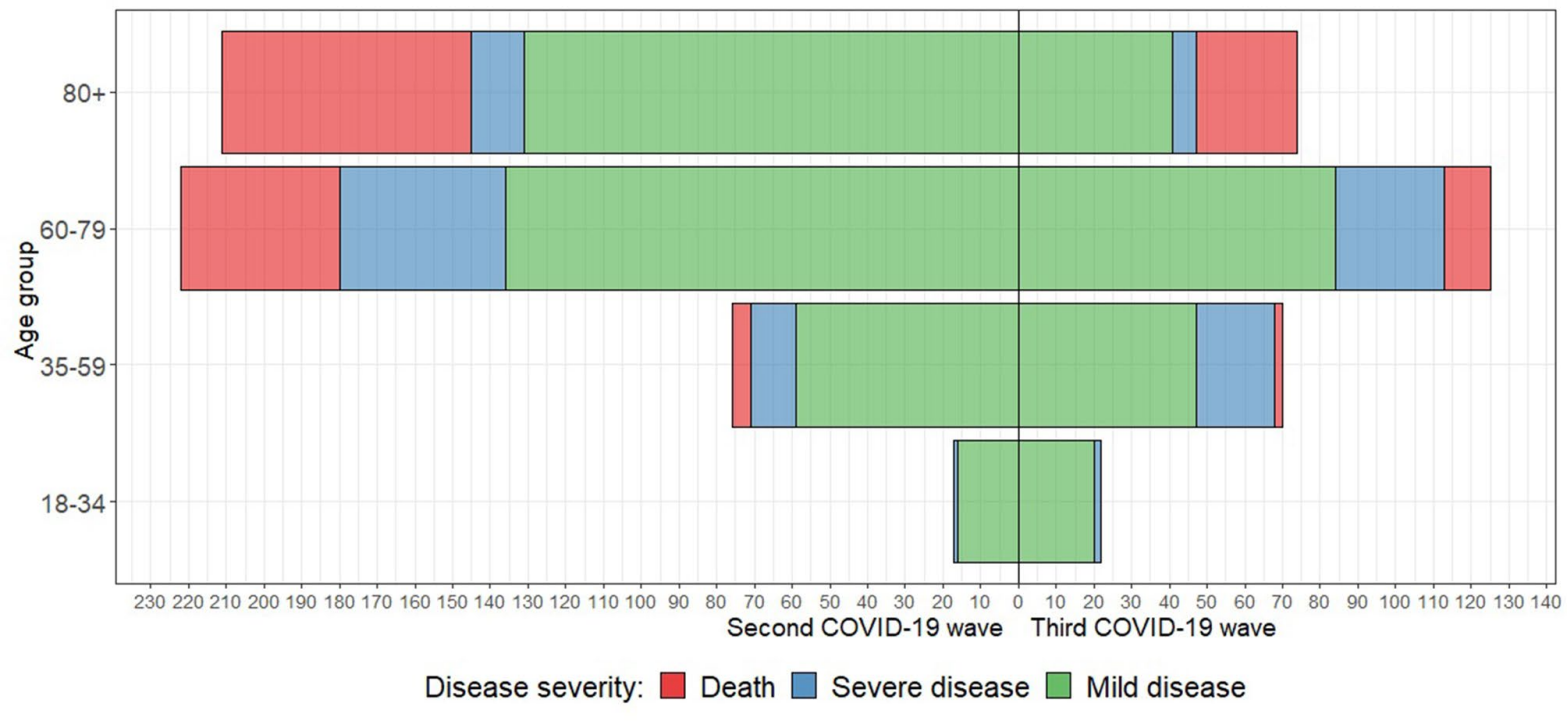
Age pyramid with differentiation by COVID-19 waves and disease severity (mild disease = WHO score 4, severe disease = WHO scores 5–7)

Figure 2 summarizes the distribution of patients between the second and third COVID-19 waves by age group and disease severity.

### COVID-19 specific drug therapy

The analysis of the distribution of COVID-19 drug therapy revealed that 32% of the patients received no specific treatment. This proportion decreased from 39.2% during the second COVID-19 wave to 17.5% during the third COVID-19 wave. Corticosteroids were the most commonly used drug group, accounting for 61.7% overall, of which 35.1% were administered as monotherapy and 25.4% in combination with remdesivir; 95.5% of the patients received corticosteroids concomitantly with remdesivir. The proportion of combination therapy with corticosteroids and remdesivir increased significantly from 17.3% during the second COVID-19 wave to 39.9% during the third COVID-19 wave. Remdesivir as monotherapy was used in 5.2% of cases, with a similar proportion during both waves. Of the 267 patients treated with remdesivir, the exact onset of symptoms was unknown in 72 cases (27%). The median duration of symptoms before the first remdesivir infusion was 5 days (Table 1). Ninety-one patients (50.5%) received remdesivir starting on the day of hospital admission and 83 (46.1%) starting on the following 2 days. The remaining 15 patients received remdesivir within 6 days of admission.

Monoclonal antibodies were used in only 2.3% of hospitalized patients (20 cases), following a case-by-case decision. Based on these results, the evaluation of patients treated with corticosteroids and/or remdesivir was the focus of further analysis. The demographic and clinical characteristics of the groups before and after PS matching are shown in the Supplement (Table S1).

### Time to clinical improvement and mortality rates

**Fig. 3:**
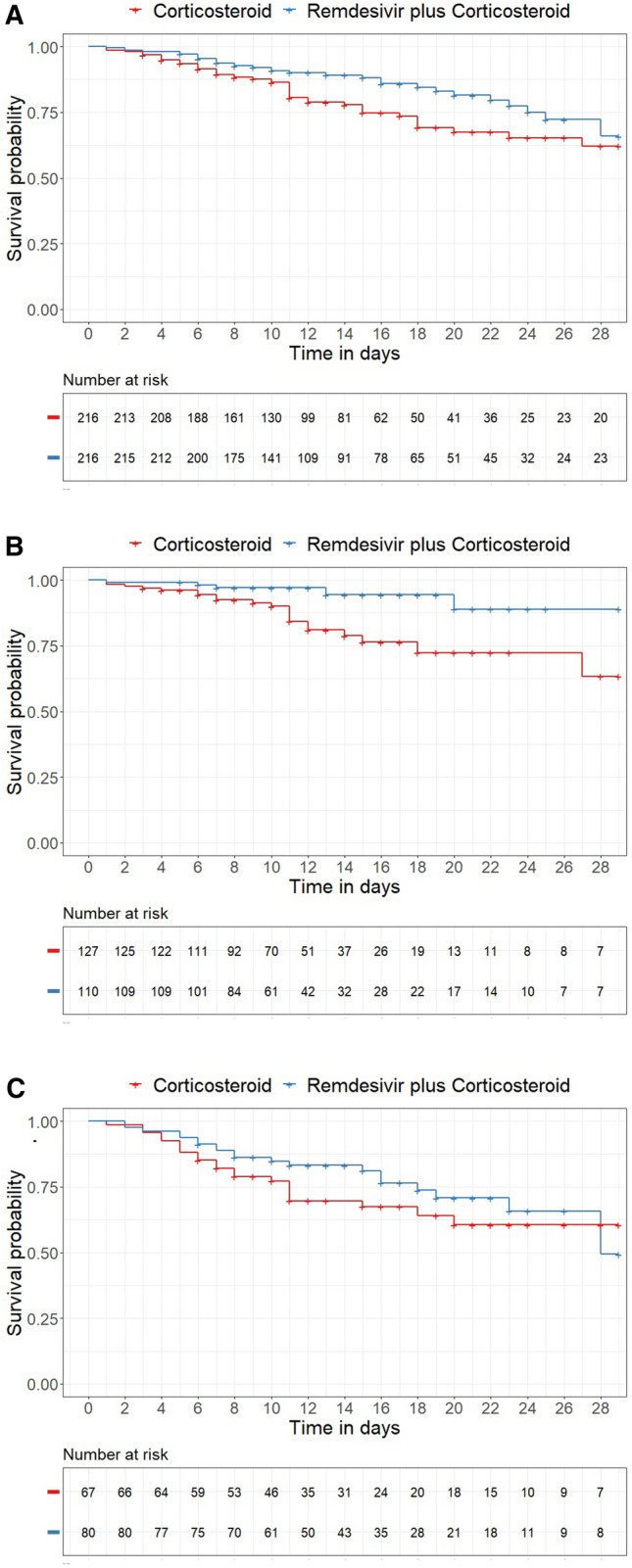
Kaplan–Meier survival curves, differentiated by therapy group (corticosteroid vs. corticosteroid–remdesivir). Panel A: All patients (HR 0.60 [95% CI 0.39–0.95], *P* = 0.027). Panel B: Patients with low-flow oxygen therapy (HR 0.26 [95% CI 0.10–0.69], *P* = 0.007). Panel C: Patients with high-flow oxygen therapy, HR 0.75 [95% CI 0.41–1.37], *P* = 0.341)

Of 432 matched cases, 150 (69.4%) patients in the corticosteroid group and 163 (75.5%) in the corticosteroid-remdesivir group were discharged from the hospital within 28 days, with a median time to clinical improvement of 13 days in both the corticosteroid group (95% CI 12–15) and the corticosteroid–remdesivir group (95% CI 11–15). Cox regression analysis showed no advantage of corticosteroid–remdesivir treatment (HR 0.97 [95% CI: 0.77–1.21], *P* = 0.762). There was also no significant difference in remdesivir treatment among patients treated in the normal care unit versus the intensive care unit (ICU) and among patients receiving low- versus high-flow oxygen therapy (Table 2). Because of the small number of cases, evaluating subgroups with different oxygen requirements was not possible. Patients in the corticosteroid group had a 28-day mortality rate of 22.2% (48 deaths) compared with 14.8% (32 deaths) in the corticosteroid–remdesivir group. In Cox regression analysis, corticosteroid–remdesivir treatment was associated with significant benefits (HR 0.60 [95% CI 0.39–0.95], *P* = 0.03). The Kaplan–Meier survival curves are shown in Fig. 3.

**Table 2:** Clinical outcomes in the study population, including subgroups

Time to clinical improvement—primary outcome at day 28									Time to death—secondary outcome at day 28					
Number of patients (*n*)			Recoveries by day 28 (*n*)		Median time to recovery in days (95% CI)		Hazard ratio [HR] (95% CI, *P* value)	Odds ratio [OR] by day 28 (95% CI, *P* value)	No of deaths by day 28		Median time to death in days (95% CI)		Hazard ratio [HR] (95% CI, *P* value)	Odds ratio [OR] by day 28 (95% CI, *P* value)
Corticosteroid versus combination of corticosteroid and remdesivir														
All patients	216	216	150	163	13 (12–15)	13 (11–15)	0.97 (0.77–1.21, * P* = 0,762)	1.35 (0.887–2.07, *P* = 0.162)	48	32	-	-	0.60 (0.39–0.95, *P* = 0.027)	0.609 (0.369–0.993, *P* = 0.048)
Subgroup, oxygen administration														
Low-flow oxygen	127	110	100	98	11 (10–13)	11 (9–11)	1.05 (0.79–1.39, * P* = 0.739)	2.2 (1.079–4.74, *P* = 0.035)	21	5	-	-	0.26 (0.10–0.69, *P* = 0.007)	0.24 (0.078–0.615, *P* = 0.006)
High-flow oxygen	67	80	39	53	17 (15–22)	16 (14–21)	1.05 (0.69–1.59, * P* = 0.806)	1.41 (0.721–2.768, *P* = 0.316)	22	21	-	28 (28–N/A)	0.75 (0.41–1.37, *P* = 0.341)	0.73 (0.355–1.485, *P* = 0.383)
Subgroup, ward type														
Normal ward	167	151	125	130	12 (11–13)	11 (10–12)	1.09 (0.85–1.39, * P* = 0.491)	2.08 (1.178–3.76, *P* = 0.013	31	13	-	-	0.45 (0.23–0.86, *P* = 0.015)	0.413 (0.201–0.807, *P* = 0.012)
Intensive care unit (ICU)	49	65	25	33	21 (17–28)	22 (17–NA)	0.87 (0.52–1.46, * P* = 0.588)	0.99 (0.47–2.08, *P* = 0.979)	17	19	-	-	0.74 (0.39–1.435, *P* = 0.379)	0.77 (0.35–1.729, *P* = 0.535)
Subgroup, COVID-19 waves														
Second wave	136	91	85	61	15 (15–19)	16 (14–21)	0.86 (0.62–1.20, *P* = 0.378)	1.22 (0.70–2.14, *P* = 0.485)	38	14	-	-	0.48 (0.26–0.88, *P* = 0.0183)	0.47 (0.23–0.91, *P* = 0.029)
Third wave	78	116	63	93	9 (8–11)	11 (10–13)	0.82 (0.60–1.13, *P* = 0.235)	0.96 (0.46–1.97, *P* = 0.09)	10	18	-	28 (28–N/A)	1.09 (0.50–2.36, *P* = 0.827)	1.25 (0.55–2.97, *P* = 0.601)
No additional COVID-19 drug therapy (SOC) versus remdesivir therapy														
All patients	44	44	30	39	10 (6–16)	10 (9–13)	1.00 (0.62–1.62, *P* = 0.993)	3.64 (1.242–12.3, *P* = 0.024)	12	3	19 (16–N/A)	-	0.20 (0.06–0.72, *P* = 0.006)	0.195 (0.042–0.675, *P* = 0.017)
Subgroup, oxygen administration														
Low-flow oxygen	36	31	25	28	10 (6–22)	9 (8–14)	1.19 (0.69–2.05, *P* = 0.531)]	4.107 (1.131–19.705, *P* = 0.045)	9	2	-	-	0.23 (0.05–1.09, *P* = 0.04)	0.207 (0.03–0.892, *P* = 0.056)
Subgroup, ward type														
Normal ward	43	44	30	39	9 (6–16)	10 (9–13)	0.99 (0.61–1.59, * P* = 0.968)	3.38 (1.139–11.501, *P* = 0.035)	11	3	19 (16–N/A)	-	0.22 (0.06–0.79, *P* = 0.01)	0.213 (0.045–0.748, *P* = 0.026)

Significant differences associated with remdesivir treatment were found for patients treated in the normal ward (HR 0.45 [95% CI 0.23–0.86], *P* = 0.015) and for patients receiving low-flow oxygen therapy (HR 0.26 [95% CI 0.10–0.69], *P* = 0.007). As disease severity increased (ICU treatment or high-flow oxygen therapy), there was no significant difference in the benefit of additional treatment with remdesivir.

### COVID-19 therapy with remdesivir compared with SOC

**Fig. 4:**
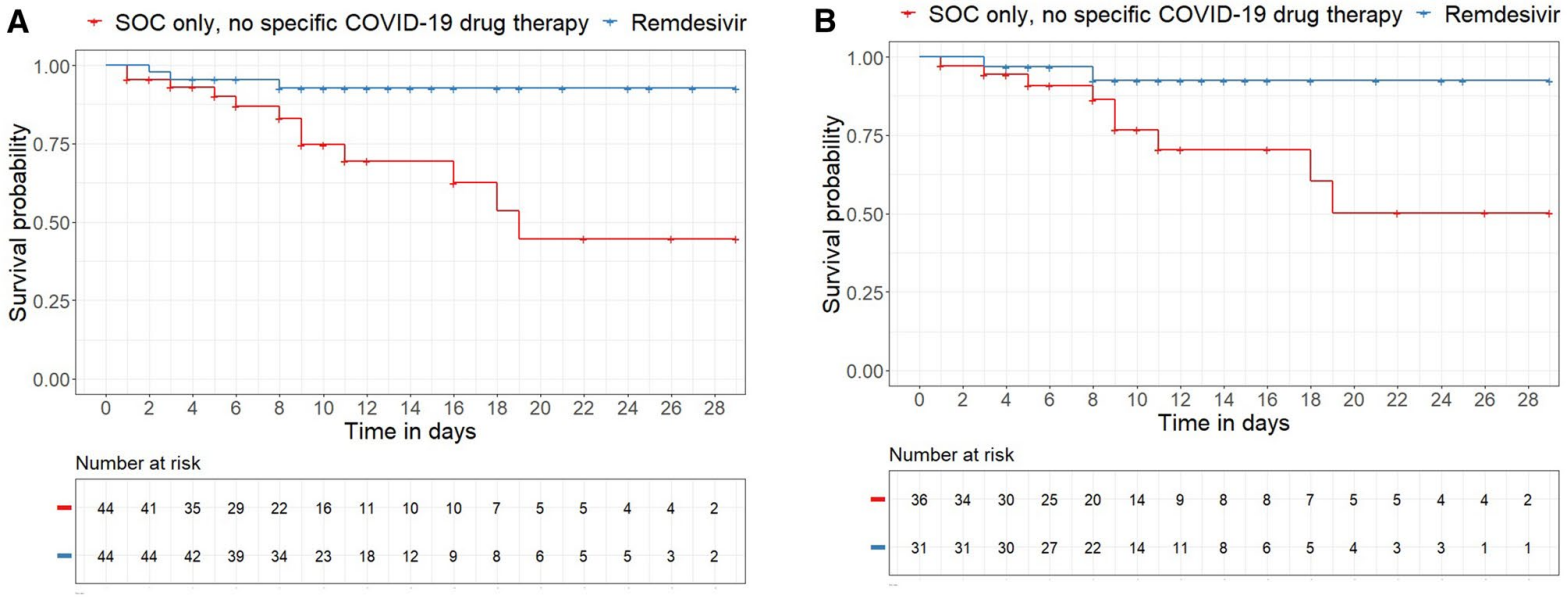
Kaplan–Meier survival curves, differentiated by therapy group (SOC vs. remdesivir). Panel A: All patients (HR 0.20 [95% CI 0.06–0.72], *P* = 0.006). Panel B: Patients with low-flow oxygen therapy (HR 0.23 [95% CI 0.05–1.00], P = 0.04)

We also performed a subgroup analysis to evaluate the efficacy of remdesivir alone, without additional administration of glucocorticoids, compared with SOC. The demographic and clinical characteristics of the subgroups before and after PS matching are shown in the Supplement (Table S2). Clinical improvement was achieved in 88.6% of the patients who received remdesivir monotherapy compared with 68.2% of the patients with SOC, demonstrating the treatment benefits of remdesivir therapy (OR 3.64 [95% CI 1.242–12.3], *P* = 0.024). However, the median time to clinical improvement was comparable at 10 days in both the non-remdesivir group (95% CI 6–16) and the remdesivir group (95% CI 9–13). Cox regression analysis showed no benefits of remdesivir treatment, with a HR of 1.002 (95% CI 0.62–1.62, *P* = 0.993). The reduction in mortality from 27.3% in patients with SOC to 6.8% in patients treated with remdesivir was significant (OR 0.195 [95% CI 0.042–0.675], *P* = 0.017). The median time to death could only be determined in patients without remdesivir therapy, and it was 19 days (95% CI 16–N/A). In Cox regression analysis, remdesivir treatment was associated with a significant survival benefit (HR 0.20 [95% CI 0.06–0.72], *P* = 0.006). The Kaplan–Meier survival curves are shown in Fig. 4.

Significant differences in mortality rates were also seen in the patients remaining in the normal ward (HR 0.22 [95% CI 0.06–0.79], *P* = 0.01) and in those receiving low-flow oxygen therapy (HR 0.23 [95% CI 0.05–1.09], *P* = 0.04). The mortality rate of patients receiving corticosteroid plus remdesivir combination therapy was comparable at 15.4% during the second wave and 15.5% during the third wave.

## Discussion

In this retrospective monocentric study, which focused on the second and third waves of the pandemic in Germany, slightly more than two thirds of the hospitalized patients with COVID-19 (68%) received specific drug therapy, with the proportion of patients without specific therapy halving from approximately 40% to 20% from the second to the third waves. Among the drug therapy approaches, corticosteroids (primarily dexamethasone) were used most frequently, accounting for more than 60% of the cases. Among these, the proportion of steroid administration increased from 56 to 75% of the patients from the second to the third waves, with a concomitant decrease in steroid monotherapy to one third of the patients and a doubling of the proportion of combination therapy with remdesivir during the third COVID-19 wave to 40%.

The results of clinical trials, systematic reviews and meta-analyses that investigated the administration of remdesivir in hospitalized patients remain inconsistent and continue to provide uncertainty regarding its potential benefits [20–29]. A recent meta-analysis of the efficacy and safety of remdesivir [28] summarized the clinical recovery and all-cause mortality end points from three RCTs [4, 30, 31] and one observational study [32]. According to this analysis, the rate of recovery with remdesivir therapy increased by 21% (risk ratio [RR] 1.21 [95% CI 1.08–1.35]) at day 7, by 29% (RR 1.29 [95% CI 1.22–1.37]) at day 14, and by 9% (RR 1.09 [95% CI 1.01–1.14]) at day 28; pooled estimates of all-cause mortality showed a 39% reduction in mortality risk with remdesivir therapy at day 14 (RR 0.61 [95% CI 0.46–0.79], *P* = 0.003) and a non-significant 22% reduction in all-cause mortality at day 28 (RR 0.78 [95% CI 0.59–1.03], *P* = 0.09). After exclusion of the study by Wang et al. [30] from the meta-analysis, the pooled data showed a significant 27% reduction in 28-day mortality (RR 0.73 [95% CI 0.54–0.99], *P* = 0.04) [28]. A recent RCT (PINETREE) of non-hospitalized patients at high risk for COVID-19 progression showed that a 3-day course of remdesivir resulted in an 87% lower risk of hospitalization or death than placebo [33]. In contrast to the PINETREE trial, which involved early administration of remdesivir in the outpatient setting, the randomized controlled DisCoVeRy trial found no clinical benefit from the use of remdesivir in patients who were hospitalized for COVID-19, had symptoms for more than 7 days and required oxygen support [29]. By contrast, there is reliable evidence of the clinical efficacy of dexamethasone in hospitalized patients with moderate to severe COVID-19. Compared to the group of patients receiving SOC only, a significant reduction in 28-day mortality was observed in patients with dexamethasone therapy and invasive ventilation (29.3% vs. 41.4%, RR 0.64 [95% CI 0.51–0.81]), and in those with non-invasive ventilation therapy (23.3% vs. 26.2%; RR 0.82 [95% CI 0.72–0.94]) [10]. However, no data from RCTs are available yet for the combination of corticosteroids with remdesivir [13, 34–37].

This prompted us to conduct a comprehensive retrospective analysis of the efficacy of combination therapy with corticosteroids and remdesivir compared with remdesivir monotherapy in our own setting. Clinical improvement occurred within 28 days in 69.4% of the patients receiving corticosteroids and in 75.5% of those in the corticosteroid-remdesivir group. The median time to clinical improvement of 13 days was the same in both matched therapy groups; this was also demonstrated in the subgroup analysis by disease severity (i.e., treatment in normal wards vs. ICU; and low- vs. high-flow oxygen therapy). Mortality was significantly lower in the corticosteroid-remdesivir group at 14.8% compared with 22.2% in the corticosteroid group (*P* = 0.048). The survival benefit was also clearly evident in the Cox regression analysis (HR 0.60, *P* = 0.03) for combined corticosteroid-remdesivir treatment. Further differences were seen in the subgroup analysis by disease severity (normal ward: HR 0.45, *P* = 0.015; low-flow oxygen therapy: HR 0.26, *P* = 0.007). As disease severity increased (treatment in the ICU or high-flow oxygen therapy), no significant difference in mortality between the two treatment groups could be detected. These results are consistent with those of a prospective, non-randomized clinical trial from Italy of 151 COVID-19 patients (76 cases with remdesivir plus dexamethasone vs. 75 cases with dexamethasone as monotherapy), which showed that combined treatment with remdesivir plus dexamethasone reduced 30-day mortality, shortened the length of hospital stay, and prevented disease progression [37]. A Danish multi-center cohort study showed that the combination of remdesivir plus dexamethasone reduced 30-day mortality compared with standard non-pharmacologic treatment (SOC); the 30-day mortality rate of 1,694 patients receiving additional remdesivir plus dexamethasone decreased to 12.6% compared with 19.7% in 1,053 patients in the SOC group (OR 0.47) [34].

We also performed a subgroup analysis to evaluate the efficacy of remdesivir alone, without additional administration of glucocorticoids. Clinical improvement was achieved in 88.6% of our patients who received remdesivir monotherapy compared with 68.2% of the patients with SOC only, demonstrating the treatment benefits of remdesivir therapy (OR 3.64, *P* = 0.024). However, the median time to clinical improvement was comparable at 10 days in both the group without remdesivir treatment and the remdesivir group. The reduction in mortality from 27.3% in the patients without specific therapy to 6.8% in those treated with remdesivir was significant (OR 0.195, *P* = 0.017). In Cox regression analysis, remdesivir treatment was associated with significant benefits (HR 0.20, *P* = 0.006). Significant differences in mortality were also seen in the patients remaining in the normal ward (HR 0.22, *P* = 0.01) and in those receiving low-flow oxygen therapy (HR 0.23, *P* = 0.04). The mortality rate of the patients receiving corticosteroid plus remdesivir combination therapy was comparable at 15.5% during the third wave and 15.4% during the second wave. By contrast, the mortality rate for corticosteroid monotherapy decreased from 27.9% during the second COVID-19 wave to 12.8% during the third COVID-19 wave. In particular, the administration of methylprednisolone as pulse therapy (IV, 250 mg/day for 3 days) has been established in the treatment of severely ill COVID-19 patients during the third wave of COVID-19 and has led to a significant reduction in mortality compared with standard treatment [38].

The third COVID-19 wave in Germany was mainly driven by the emerging SARS-CoV-2 alpha variant (B.1.1.7), which has a higher reproductive number compared with the wild type virus [39]. For this reason, the federal government in Germany extended nationwide lockdown measures until early May 2021, resulting in a uniform decline in the number of infections in all states [40]. These measures and the vaccination of the most vulnerable groups, particularly the elderly, against SARS-CoV-2, which began in late December 2020, resulted in the containment of rising infection rates during the third wave of COVID and, consequently, a decrease in the hospitalization rates of the most vulnerable groups. Another striking feature of hospitalized patients during the third COVID-19 wave was the marked decrease in chronic diseases, such as hypertension, cardiovascular disease, diabetes, renal disease, and dementia, being associated with potential complications of COVID-19 and increased disease severity [41–44]. The likelihood of hospitalization because of COVID-19 was very high in patients with pre-existing conditions. The known risk factors for severe disease are older age, hypertension, cardiovascular and chronic lung disease, diabetes, immunodeficiency, smoking, and male sex [42–46]. During the study period, 74.1% of the patients with COVID-19 were admitted with cardiovascular disease. Among them, the diagnosis of hypertension was very frequent (67.3%). Metabolic disorders, such as diabetes (30.5%), obesity (15.4%), and renal disease (30.9%) also dominated. Although 65.1% of the patients had mild or moderate COVID-19 (WHO scores 3–4), there was a high proportion of patients with severe (scores 5–7) and fatal courses (score 8). Mortality in our study population was 18.2%, which is comparable to in-hospital mortality in other tertiary care centers in France (18.1%) [47] and Germany (17%) [48], but significantly lower than that in the UK, where an in-hospital mortality of 26% has been reported [45].

Our study has several limitations. First, it is a retrospective, observational study at a single center, and despite the use of propensity score matching, we cannot exclude the possibility that there are other unmeasured confounders that could bias our estimates of the treatment effect. For example, remdesivir has to be administered intravenously, for which there are hardly any outpatient structures in Germany, and some of the patients did not receive remdesivir because the disease was already too advanced. Late presentation may have influenced selection towards a severe course in several ways. Second, despite the large size of the cohort studied (*n* = 839 fully evaluable patients), subgroups are relatively small, especially when the analysis was restricted to matched groups, and the validity of our results needs to be confirmed in larger studies. However, although the results of a retrospective cohort cannot replace the results of RCTs, these data may help answer a question for which RCT data are incomplete, namely whether the use of remdesivir is associated with lower mortality in hospitalized patients with COVID-19.

## Conclusion

In our cohort of COVID-19 patients with only mild disease (low-flow oxygen therapy and treatment in a normal ward) who received corticosteroids and/or remdesivir in addition to SOC, early administration of remdesivir was associated with a measurable survival benefit.

## Supplementary Information

Below is the link to the electronic supplementary material.

Supplementary file1 (DOCX 20 KB)
