# Supplementary material for: Clinical outcomes of hospitalized COVID-19 patients treated with remdesivir: a retrospective analysis of a large tertiary care center in Germany
Source: Infection. 2022 May 12;51(1):97–108. doi: 10.1007/s15010-022-01841-8 (PMC9098143; doi:10.1007/s15010-022-01841-8)
Supplement: Supplementary file 1 — Supplementary file1 (DOCX 20 KB) [file 15010_2022_1841_MOESM1_ESM.docx]

**Supplement**

|  | **All patients** | | | |  | **Propensity score-matched patients** | | | |
| --- | --- | --- | --- | --- | --- | --- | --- | --- | --- |
| **Characteristics** | **Corticosteroid** | **Corticosteroid plus remdesivir** | ***P* value** | **SMD** |  | **Corticosteroid** | **Corticosteroid plus remdesivir** | ***P* value** | **SMD** |
| **Number of patients** (n) | 299 | 216 |  |  |  | 216 | 216 |  |  |
| **Age** (years, mean, SD) | 72.05 (14.96) | 68.38 (15.88) | 0.008 | 0.238 |  | 70.93 (15.67) | 68.38 (15.88) | 0.094 | 0.161 |
| **Male gender** (%) | 180 (60.2) | 131 (60.6) | 0.991 | 0.009 |  | 134 (62.0) | 131 (60.6) | 0.843 | 0.029 |
| **BMI** (mean, SD) | 29.48 (6.49) | 29.25 (6.47) | 0.748 | 0.035 |  | 29.44 (6.43) | 29.25 (6.47) | 0.804 | 0.029 |
| **Oxygen administration** (%) | | | | | | | | | |
| No supplementation of oxygen | 14 (4.7) | 6 (2.8) | 0.033 | 0.265 |  | 8 (3.7) | 6 (2.8) | 0.294 | 0.186 |
| Low-flow oxygen | 184 (61.5) | 110 (50.9) |  |  |  | 127 (58.8) | 110 (50.9) |  |  |
| High-flow oxygen | 82 (27.4) | 80 (37.0) |  |  |  | 67 (31.0) | 80 (37.0) |  |  |
| Invasive vent./ECMO | 19 (6.4) | 20 (9.3) |  |  |  | 14 (6.5) | 20 (9.3) |  |  |
|  | | | | | | | | | |
| **Respiratory rate/min** | 22.25 (5.36) | 21.37 (6.25) | 0.131 | 0.152 |  | 22.03 (5.37) | 21.37 (6.25) | 0.295 | 0.114 |
| **Laboratory results** (mean, SD) | | | | | | | | | |
| Estimated glomerular filtration rate (mL/min/1.73 m2) | 64.81 (34.02) | 74.63 (33.73) | 0.001 | 0.290 |  | 69.29 (34.16) | 74.63 (33.73) | 0.107 | 0.157 |
| C-reactive protein (mg/L) | 98.57 (78.28) | 86.43 (128.82) | 0.191 | 0.114 |  | 91.04 (72.48) | 86.43 (128.82) | 0.651 | 0.044 |
| Leukocyte count (Gpt/L) | 7.73 (4.42) | 6.61 (4.43) | 0.005 | 0.252 |  | 6.86 (3.23) | 6.61 (4.43) | 0.509 | 0.064 |
| Platelet count (Gpt/L) | 219.58 (109.08) | 175.85 (64.02) | <0.001 | 0.489 |  | 187.18 (68.31) | 175.85 (64.02) | 0.077 | 0.171 |
| Alanine aminotransferase (µmol/L*s) | 0.67 (0.86) | 0.68 (0.57) | 0.822 | 0.025 |  | 0.63 (0.82) | 0.68 (0.57) | 0.526 | 0.074 |
| Aspartate aminotransferase (µmol/L*s) | 1.02 (1.36) | 1.00 (0.77) | 0.882 | 0.016 |  | 0.99 (0.98) | 1.00 (0.77) | 0.920 | 0.011 |
| D-dimer (µg/L) | 2590.45 (4133.68) | 2101.17 (3588.02) | 0.192 | 0.126 |  | 2094.64 (3498.27) | 2101.17 (3588.02) | 0.986 | 0.002 |
| **Comorbidities** (%) | | | | | | | | | |
| Hypertension | 217 (72.6) | 146 (67.6) | 0.260 | 0.109 |  | 156 (72.2) | 146 (67.6) | 0.345 | 0.101 |
| Cardiovascular disease | 240 (80.3) | 160 (74.1) | 0.119 | 0.148 |  | 170 (78.7) | 160 (74.1) | 0.308 | 0.109 |
| Diabetes | 110 (36.8) | 66 (30.6) | 0.168 | 0.132 |  | 73 (33.8) | 66 (30.6) | 0.537 | 0.069 |
| Obesity | 60 (20.1) | 34 (15.7) | 0.255 | 0.113 |  | 37 (17.1) | 34 (15.7) | 0.795 | 0.037 |
| Chronic kidney disease | 51 (17.1) | 28 (13.0) | 0.251 | 0.115 |  | 33 (15.3) | 28 (13.0) | 0.581 | 0.067 |
| Liver disease | 8 (2.7) | 3 (1.4) | 0.492 | 0.091 |  | 2 (0.9) | 3 (1.4) | 1.000 | 0.043 |
| Cancer | 18 (6.0) | 20 (9.3) | 0.224 | 0.122 |  | 15 (6.9) | 20 (9.3) | 0.481 | 0.085 |
| Asthma | 13 (4.3) | 14 (6.5) | 0.383 | 0.094 |  | 12 (5.6) | 14 (6.5) | 0.840 | 0.039 |
| COPD | 31 (10.4) | 22 (10.2) | 1.000 | 0.006 |  | 26 (12.0) | 22 (10.2) | 0.646 | 0.059 |
| Dementia | 39 (13.0) | 14 (6.5) | 0.023 | 0.222 |  | 16 (7.4) | 14 (6.5) | 0.850 | 0.036 |
| Delirium | 10 (3.3) | 13 (6.0) | 0.217 | 0.127 |  | 10 (4.6) | 13 (6.0) | 0.668 | 0.062 |
| ***Abbreviations:*** *BMI, body mass index; D-dimer, dimerized plasmin fragment D; COPD, chronic obstructive pulmonary disease; SD, standard deviation; SMD, standardized mean difference* | | | | | | | | | |

***Table S1****. Clinical characteristics of patients in the corticosteroid and corticosteroid-remdesivir groups before and after propensity score matching*

|  | **All patients** | | | |  | **Propensity score-matched patients** | | | |
| --- | --- | --- | --- | --- | --- | --- | --- | --- | --- |
| **Characteristics** | **SOC only** | **Remdesivir** | ***P* value** | **SMD** |  | **SOC only** | **Remdesivir** | ***P* value** | **SMD** |
| **Number of patients** (n) | 260 | 44 |  |  |  | 44 | 44 |  |  |
| **Age** (years, mean, SD) | 69.22 (17.68) | 68.75 (17.94) | 0.871 | 0.026 |  | 74.25 (15.42) | 68.75 (17.94) | 0.127 | 0.329 |
| **Male gender** (%) | 143 (55.0) | 18 (40.9) | 0.117 | 0.285 |  | 22 (50.0) | 18 (40.9) | 0.521 | 0.183 |
| **BMI** (mean, SD) | 27.81 (5.53) | 27.81 (4.33) | 0.998 | 0.001 |  | 28.05 (5.31) | 27.81 (4.33) | 0.883 | 0.049 |
| **Oxygen administration** (%) | | | | | | | | | |
| No supplementation of oxygen | 146 (56.2) | 6 (13.6) | <0.001 | 1039 |  | 5 (11.4) | 6 (13.6) | 0.356 | 0.310 |
| Low-flow oxygen | 106 (40.8) | 31 (70.5) |  |  |  | 36 (81.8) | 31 (70.5) |  |  |
| High-flow oxygen | 8 (3.1) | 7 (15.9) |  |  |  | 3 (6.8) | 7 (15.9) |  |  |
|  | | | | | | | | | |
| **Respiratory rate/min** | 18.62 (5.79) | 19.44 (4.82) | 0.453 | 0.154 |  | 19.96 (5.09) | 19.44 (4.82) | 0.700 | 0.104 |
| **Laboratory results** (mean, SD) | | | | | | | | | |
| Estimated glomerular filtration rate (ml/min/1.73 m2) | 72.26 (35.33) | 71.35 (26.92) | 0.872 | 0.029 |  | 67.29 (30.43) | 71.35 (26.92) | 0.514 | 0.141 |
| C-reactive protein (mg/L) | 46.28 (60.62) | 63.18 (70.91) | 0.107 | 0.256 |  | 45.29 (49.81) | 63.18 (70.91) | 0.185 | 0.292 |
| Leukocyte count (Gpt/L) | 8.73 (12.25) | 5.90 (2.51) | 0.133 | 0.319 |  | 5.76 (2.64) | 5.90 (2.51) | 0.793 | 0.057 |
| Platelet count (Gpt/L) | 232.27 (114.56) | 191.42 (67.22) | 0.024 | 0.435 |  | 190.72 (90.52) | 191.42 (67.22) | 0.968 | 0.009 |
| Alanine aminotransferase (µmol/L*s) | 0.55 (0.42) | 0.51 (0.34) | 0.660 | 0.095 |  | 0.43 (0.22) | 0.51 (0.34) | 0.300 | 0.268 |
| Aspartate aminotransferase (µmol/L*s) | 0.82 (0.91) | 0.76 (0.45) | 0.750 | 0.074 |  | 0.67 (0.37) | 0.76 (0.45) | 0.384 | 0.223 |
| D-dimer (µg/L) | 2937.71 (4858.96) | 1927.19 (3502.03) | 0.238 | 0.239 |  | 1741.67 (2025.30) | 1927.19 (3502.03) | 0.806 | 0.065 |
| **Comorbidities** (%) | | | | | | | | | |
| Hypertension | 172 (66.2) | 22 (50.0) | 0.058 | 0.332 |  | 27 (61.4) | 22 (50.0) | 0.391 | 0.230 |
| Cardiovascular disease | 190 (73.1) | 25 (56.8) | 0.044 | 0.346 |  | 30 (68.2) | 25 (56.8) | 0.378 | 0.236 |
| Diabetes | 73 (28.1) | 6 (13.6) | 0.067 | 0.361 |  | 11 (25.0) | 6 (13.6) | 0.280 | 0.291 |
| Obesity | 33 (12.7) | 3 (6.8) | 0.388 | 0.199 |  | 4 (9.1) | 3 (6.8) | 1000 | 0.084 |
| Chronic kidney disease | 41 (15.8) | 3 (6.8) | 0.184 | 0.286 |  | 5 (11.4) | 3 (6.8) | 0.711 | 0.159 |
| Liver disease | 7 (2.7) | 0 (0.0) | 0.577 | 0.235 |  | 0 (0.0) | 0 (0.0) | NA | <0.001 |
| Cancer | 33 (12.7) | 4 (9.1) | 0.670 | 0.116 |  | 5 (11.4) | 4 (9.1) | 1000 | 0.075 |
| Asthma | 6 (2.3) | 5 (11.4) | 0.011 | 0.365 |  | 3 (6.8) | 5 (11.4) | 0.711 | 0.159 |
| COPD | 20 (7.7) | 3 (6.8) | 1.000 | 0.034 |  | 5 (11.4) | 3 (6.8) | 0.711 | 0.159 |
| Dementia | 36 (13.8) | 6 (13.6) | 1000 | 0.006 |  | 7 (15.9) | 6 (13.6) | 1000 | 0.064 |
| Delirium | 15 (5.8) | 1 (2.3) | 0.551 | 0.179 |  | 2 (4.5) | 1 (2.3) | 1.000 | 0.125 |
| ***Abbreviations:*** *BMI, body mass index; D-dimer, dimerized plasmin fragment D; COPD, chronic obstructive pulmonary disease; SOC, standard of care (no additional drug therapy für COVID-19); SD, standard deviation; SMD, standardized mean difference* | | | | | | | | | |

***Table S2****. Baseline characteristics of patients in the remdesivir and SOC groups before and after propensity score matching*
